# Advancing diabetes treatment: the role of mesenchymal stem cells in islet transplantation

**DOI:** 10.3389/fimmu.2024.1389134

**Published:** 2024-03-28

**Authors:** Lisha Mou, Tony Bowei Wang, Xinyu Wang, Zuhui Pu

**Affiliations:** ^1^ Department of Endocrinology, Institute of Translational Medicine, The First Affiliated Hospital of Shenzhen University, Shenzhen Second People’s Hospital, Shenzhen, Guangdong, China; ^2^ MetaLife Lab, Shenzhen Institute of Translational Medicine, Shenzhen, Guangdong, China; ^3^ Biology Department, Skidmore College, Saratoga Springs, NY, United States; ^4^ Imaging Department, The First Affiliated Hospital of Shenzhen University, Shenzhen Second People’s Hospital, Shenzhen, Guangdong, China

**Keywords:** diabetes, islets, islet transplantation, MSC, immunomodulation, single-cell RNA sequencing, artificial intelligence, regenerative medicine

## Abstract

Diabetes mellitus, a prevalent global health challenge, significantly impacts societal and economic well-being. Islet transplantation is increasingly recognized as a viable treatment for type 1 diabetes that aims to restore endogenous insulin production and mitigate complications associated with exogenous insulin dependence. We review the role of mesenchymal stem cells (MSCs) in enhancing the efficacy of islet transplantation. MSCs, characterized by their immunomodulatory properties and differentiation potential, are increasingly seen as valuable in enhancing islet graft survival, reducing immune-mediated rejection, and supporting angiogenesis and tissue repair. The utilization of MSC-derived extracellular vesicles further exemplifies innovative approaches to improve transplantation outcomes. However, challenges such as MSC heterogeneity and the optimization of therapeutic applications persist. Advanced methodologies, including artificial intelligence (AI) and single-cell RNA sequencing (scRNA-seq), are highlighted as potential technologies for addressing these challenges, potentially steering MSC therapy toward more effective, personalized treatment modalities for diabetes. This review revealed that MSCs are important for advancing diabetes treatment strategies, particularly through islet transplantation. This highlights the importance of MSCs in the field of regenerative medicine, acknowledging both their potential and the challenges that must be navigated to fully realize their therapeutic promise.

## 1 Introduction

Diabetes mellitus has emerged as a significant chronic disease worldwide that imposes substantial social and economic burdens. In 2021, around 537 million adults aged 20-79 were reported to have diabetes (1), including approximately 8.4 million with type 1 diabetes (T1D) (2). T1D, a chronic autoimmune disease, necessitates lifelong management via daily insulin injections or continuous infusion through a pump (3). Despite advancements, precise insulin dosage control remains challenging for some patients, leading to the risk of hypoglycemia and further complicating treatment (4). The FDA approval of Lantidra, the initial allogeneic pancreatic islet cell therapy product derived from deceased donors, marked a significant innovation in islet transplantation, offering new hope for T1D patients struggling with severe hypoglycemia despite intensive diabetes management (5, 6). This development not only signifies a breakthrough in transplantation technology but also provides an effective alternative treatment for adult T1D patients who cannot reach the desired glycated hemoglobin levels.

Islet transplantation, as an innovative treatment, offers T1D patients the potential to restore endogenous insulin production, reducing reliance on exogenous insulin and preventing long-term complications (7). This minimally invasive method has shown effectiveness beyond traditional treatments (8), with some centers reporting over 50% insulin independence after five years (9, 10). However, challenges remain, including donor cell scarcity, substantial cell loss post-transplantation due to immediate blood-mediated inflammatory reactions, hypoxia, and ischemia-reperfusion injury, as well as complications from immunosuppressants (11). Consequently, achieving optimal glucose control often necessitates multiple transplants.

Recent strides in regenerative medicine have identified mesenchymal stem cells (MSCs) as key players in overcoming these obstacles (12). Due to their multipotency, immunomodulatory properties, and low immunogenicity, MSCs derived from bone marrow, adipose tissue, and umbilical cord blood have demonstrated potential for enhancing islet graft survival, modulating immune responses, and promoting tissue repair and angiogenesis (13). This has been evidenced by their capacity to differentiate into different cell types, such as cells that produce insulin, and their secretion of a vast array of regenerative factors, making them promising candidates for co-transplantation with pancreatic islets (14). The immunomodulatory properties of MSCs, which are pivotal for modulating immune responses and fostering tissue repair, have been extensively documented, suggesting a significant reduction in transplant rejection risk (15, 16). Additionally, the therapeutic versatility of MSCs has been explored in various disease contexts, including cardiovascular diseases and liver injury, further supporting their broad potential in regenerative medicine.

Moreover, the exploration of MSC-derived exosomes represents an advancement in therapeutic strategies for pancreatic islet transplantation, highlighting a new direction in MSC-based treatments (17). These exosomes, as carriers of regenerative and immunomodulatory factors, present a novel approach to enhancing the microenvironment of transplanted islets, potentially reducing immunological rejection and supporting islet cell survival and function. This advancement in MSC-derived exosome research illustrates the ongoing innovation in treatment methodologies, aiming to address the complex challenges of islet transplantation in diabetes management.

The comparison between MSCs, induced pluripotent stem cells (iPSCs), and embryonic stem cells (ESCs) highlights their distinct roles in diabetes treatment. MSCs are favored for their immunomodulatory properties and minimal tumorigenic risk, presenting a safer option for clinical applications. Unlike MSCs, iPSCs and ESCs possess greater pluripotency, enabling the generation of a broader range of cell types. However, this approach is associated with increased ethical concerns for ESCs and increased tumorigenic potential for both iPSCs and ESCs (18, 19). Compared to their broader differentiation capabilities, the unique advantages of MSCs in regenerative medicine, particularly in mitigating autoimmune responses in diabetes, are the heightened risks associated with iPSCs and ESCs.

To enhance our understanding of MSC therapies, particularly in the realms of diabetes treatment and islet transplantation, advanced technologies such as artificial intelligence (AI) and single-cell RNA sequencing (scRNA-seq) offer groundbreaking approaches (20, 21). These innovations allow for the precise characterization of MSCs and in-depth analysis of their molecular behaviors, which is crucial for developing personalized, effective therapies. By incorporating findings from a recent study that utilized scRNA-seq to analyze immune heterogeneity in mouse models of islet transplantation, we obtained valuable insights into the immune mechanisms that may affect graft survival (22). The insights of this study into the transcriptomics of islet grafts underscore the potential of scRNA-seq for identifying key factors influencing the success of MSC therapies, highlighting the importance of these advanced methodologies in advancing personalized medicine and improving diabetes care strategies.

This review not only highlights the potential of MSCs in enhancing diabetes treatment through islet transplantation but also addresses persistent challenges such as MSC heterogeneity and the need for therapeutic optimization. This finding underscores the promise of advanced methodologies such as AI and scRNA-seq for overcoming these hurdles, suggesting that MSC therapy could offer more effective, personalized diabetes treatment solutions in the future.

## 2 Background on MSCs

### 2.1 Therapeutic molecules and mechanisms of MSCs

MSCs, which are derived from bone marrow, adipose tissue, the umbilical cord, and the gingiva, play a crucial role in regenerative medicine due to their unique ability to differentiate into multiple cell types essential for tissue repair and regeneration (23). The significant immunomodulatory effects of MSCs, which impact both the innate and adaptive immune systems through the secretion of bioactive factors with immunosuppressive and anti-inflammatory properties, underscore their importance (24). These cells can differentiate into a variety of different cell types is also important, especially when addressing T1D and enhancing pancreatic islet transplantation outcomes by supporting graft survival, modulating immune responses, and promoting tissue repair and angiogenesis.

### 2.2 MSC-derived extracellular vesicles (EVs): broadening therapeutic horizons

The potential of MSC-derived EVs extends beyond diabetes to conditions such as ischemic stroke and osteoarthritis, demonstrating the wide range of applications of MSCs (25). Clinical studies have confirmed the ability of MSCs to evolve into insulin-producing cells and secrete healing factors, positioning them as key players in T1D treatment (26). Furthermore, engineered MSC-derived EVs are being developed to enhance regenerative efficacy, overcoming natural limitations (27). Proteomic analyses of MSC exosomes from various sources have revealed that shared mechanisms, notably extracellular matrix interactions, are crucial for their regenerative impact (28). The clinical application of umbilical cord MSCs highlights the ongoing promise of MSC-based therapies in diabetes management and beyond (26).

## 3 MSCs in improving islet transplantation outcomes

In islet transplantation for diabetes treatment, MSCs play a vital role by safeguarding islet cells and improving the outcomes of both allo- and xenotransplantation (29–31). The broad array of secreted molecules, including growth factors and immunomodulatory agents, contributes to enhancing transplantation efficacy and survival (32). During regeneration, these secretory factors facilitate tissue remodeling and promote cellular homeostasis. This multifaceted action of MSCs addresses key challenges in islet transplantation, including reducing graft rejection and improving graft performance, thus enhancing the efficacy of diabetes treatments (33, 34). Moreover, the potential of MSCs for novel therapeutic strategies marks an advancement in diabetes management, suggesting that MSCs are important elements in both regenerative medicine and autoimmune therapy. The role of MSCs in enhancing outcomes in diabetes patients through islet transplantation is critical, as they emphasize the broad therapeutic potential of these cells and open new paths for diabetes care advancements.

The exploration of the use of MSCs in diabetes treatment, particularly through islet transplantation, highlights their potential for advancing therapeutic strategies. MSCs are at the forefront of regenerative medicine and autoimmune therapy, offering innovative approaches to improve patient outcomes in diabetes care. Their broad therapeutic capabilities and potential for new pathways in diabetes management underscore their role in the field.

The integration of MSCs into pancreatic islet transplantation protocols has been the focus of numerous studies, revealing substantial improvements in transplantation outcomes (34). These cells have demonstrated the capacity to enhance both the engraftment and survival rates of transplanted islets as well as their long-term functionality, which is important for successful transplantation. MSCs exert beneficial effects through immunomodulatory effects, mitigating immune-mediated rejection and autoimmune attacks. Additionally, MSCs promote a supportive microenvironment for islets by stimulating angiogenesis and tissue repair, addressing the challenges of islet transplantation and advancing diabetes treatment modalities.

### 3.1 The protective role of MSCs during islet isolation and culture

Its ability to protect islet vitality during isolation and culture is crucial because it can mitigate hypoxia and inflammatory stress, which are key factors in islet impairment. Coculturing islets with MSCs not only preserves islet functionality but also improves transplantation results by maintaining insulin secretion and cell vitality. The role of MSCs in improving the internal microenvironment for T1D treatment has been well studied. MSCs contribute to protecting islet vitality during isolation and culture and in mitigating hypoxia and inflammatory stress, which are key factors in islet impairment (29, 35). Coculturing islets with MSCs not only preserves islet functionality but also improves transplantation results by maintaining insulin secretion and cell vitality (29, 35).

### 3.2 Enhancing islet transplantation through MSC-driven immunomodulation and angiogenesis

MSCs ameliorate the internal microenvironment post-islet transplantation, reducing inflammation and improving patient outcomes (29, 35). The capacity of these cells to alleviate blood-mediated inflammatory responses post-transplant is important for T1D patients to progress toward insulin independence. By mitigating transplant-related stress and curtailing β-cell damage, MSCs play a critical role. Furthermore, the secretion of soluble immunomodulatory factors crucially suppresses immune rejection, fostering graft tolerance.

The synergistic effect of MSCs in islet transplantation is multifaceted, emphasizing not only their anti-inflammatory effects but also their critical contribution to angiogenesis (36). After isolation, islets undergo vascularization loss, increasing susceptibility to stressors such as instant blood-mediated immune reactions, hypoxia, and ischemia-reperfusion injury, which can lead to apoptosis and necrosis. MSCs facilitate rapid revascularization; secrete factors such as VEGF (37), Ang-1 (38), bFGF (32), KGF (39), IGF-1, IGF-2, and HGF; and are important for angiogenesis and enhancing islet graft survival and functionality. MSCs can promote angiogenesis by activating AKT/MAPK signaling and upregulating VEGFR signaling (40). MSCs can also perform angiogenic modulation through complex interactions between bioactive molecules carried by EVs, such as microRNAs (40). Despite the inherent challenges of MSC use, such as limited proliferative capacity and heterogeneity, ongoing studies highlight the capacity of MSCs to support new blood vessel formation, ensuring that transplanted islets receive necessary nourishment and oxygen. Along with the ability of MSCs to promote tissue repair, this angiogenesis supports the role of MSCs in improving the outcomes of diabetes treatments through islet transplantation (41).

The multifaceted role of MSCs in islet transplantation reflects their capacity for immune modulation and support for angiogenesis and tissue repair. While their use presents challenges such as limited growth and variability, ongoing research into the origins and functionalities of MSCs continues to enhance their application in diabetes treatment. This exploration of the diverse origins of MSCs, such as bone marrow and adipose tissue, and their capacities for angiogenesis and immunosuppression contributes to understanding and leveraging their therapeutic impact in islet transplantation.

## 4 Clinical applications and insights of MSCs

The integration of MSC therapy into clinical practice presents scientific and ethical challenges, including complexities and potential risks such as immunogenicity and tumorigenicity. Examining these obstacles and emphasizing the importance of rigorous selection, ethical sourcing practices, and adherence to regulatory guidelines will ensure safety and efficacy. This highlights the necessity of continuous research, ethical deliberation, and regulatory updates to optimize MSC therapies for diverse conditions, particularly diabetes treatment through islet transplantation.

Despite the promising potential of MSCs in regenerative medicine and diabetes treatment, MSCs face significant challenges that hinder their clinical translation. These include their inherent heterogeneity, which complicates the consistency of therapeutic outcomes, and concerns about immunogenicity that may elicit immune responses in recipients (42). Additionally, the potential for MSCs to contribute to tumorigenesis remains a critical area of investigation (43). It is important to critically evaluate these challenges, drawing upon data from past clinical trials and current research, to provide a comprehensive understanding of the limitations and barriers facing the use of MSCs in islet transplantation. This underscores the complexity of translating MSC therapy from bench to bedside and emphasizes the need for continued research to overcome these obstacles.

The ethical implications of MSC sourcing involve considerations such as donor consent, particularly for MSCs derived from human tissues (44). Ethical sourcing ensures that donors are fully informed and agree to the use of their cells for research or therapeutic purposes. Regulatory policies for MSC therapies, governed by agencies such as the FDA and the European Medicines Agency, focus on ensuring the safety, efficacy, and quality of MSC-based products (45). These regulations necessitate rigorous clinical trials and manufacturing standards to prevent risks such as immunogenicity and tumorigenicity, aiming to safeguard patient health while fostering innovation in MSC therapy development.

## 5 Overcoming challenges and charting the future of MSC therapy in clinical applications

**Figure 1:**
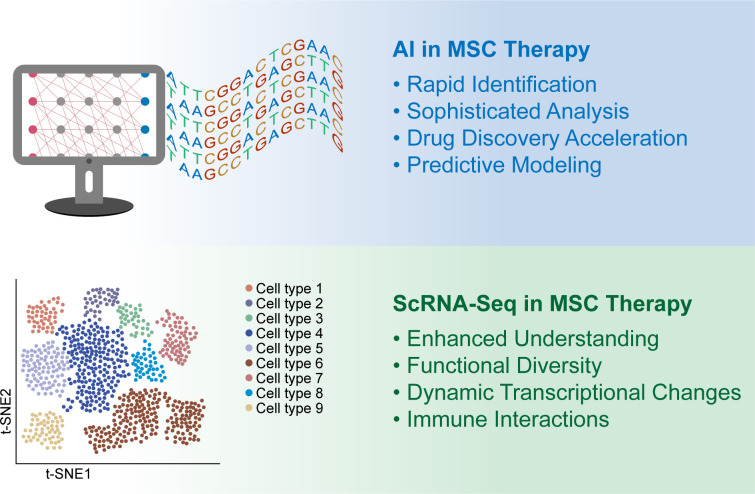
Artificial intelligence (AI) and single-cell RNA sequencing (scRNA-seq) for enhancing the clinical application of mesenchymal stem cells (MSCs) (1). The upper panel shows the AI used in MSC clinical treatment. Rapid Identification: AI has drastically accelerated the discovery of novel molecular compounds and drug targets, enhancing the development of MSC-based treatments. Sophisticated Analysis: This study utilized complex biological datasets to optimize graft longevity and functionality within islet transplantation settings. Drug Discovery Acceleration: AI contributes to drug research and development, improving the precision and efficiency of therapeutic discovery and development processes. Predictive Modeling: This method employs dynamic molecular traits for MSC therapy, including protein sequences and molecular interactions, to refine therapeutic strategies (2). The lower panel shows the results of scRNA-seq analysis of MSC therapies. Enhanced Understanding: scRNA-seq offers in-depth insights into MSC heterogeneity, enabling precise characterization and biomarker identification. Functional Diversity: This study reveals the complex roles of MSCs in development, regeneration, and pathology, facilitating the development of targeted therapies. Dynamic Transcriptional Changes: This review sheds light on MSC differentiation and the regulatory pathways involved, supporting the refinement of clinical applications. Immune Interactions: This paper describes how MSCs modulate immune cells, providing valuable information for developing MSC-based therapies for immune modulation and tissue repair.

The progression of MSCs from early preclinical studies to clinical applications illustrates both their potential and the complexities involved in MSC therapy. Despite advancements, challenges affect their clinical success (42). In diabetes treatment, especially islet transplantation, the variability and adaptability of MSCs require careful clinical evaluation. The variability of MSCs due to their different sources, preparation methods, and delivery techniques poses significant challenges to their standardization and consistency in therapies (46, 47). To minimize heterogeneity and improve the predictability of outcomes, it is important to explore strategies and potential guidelines, such as stringent cell characterization, uniform culture conditions, and standardized delivery methods (48). Emphasizing the need for comprehensive quality control and clinical protocol standardization will offer insights into advancing MSC therapies toward more reliable and effective clinical applications, thus enhancing their utility in regenerative medicine and beyond. Innovations such as AI (49) and scRNA-seq (50) are promising methods for addressing these obstacles, indicating that MSC therapy might become a standard, personalized treatment option for diabetes in the future (**Figure 1**).

### 5.1 AI in MSC clinical treatment

The integration of AI into MSC therapies for diabetes treatment is an evolving field that combines two cutting-edge scientific advancements. Although direct applications in diabetes are nascent, the synergy between the predictive capabilities of AI and the therapeutic potential of MSCs offers promising directions for more precise and customized treatments. This interdisciplinary approach aims to enhance diabetes care by leveraging AI to optimize MSC therapy outcomes, indicating a move toward more individualized and effective treatment strategies in regenerative medicine.

#### 5.1.1 Accelerating drug discovery with AI in MSC therapies

Digital technology and AI significantly influence healthcare innovation, particularly in drug research and development (51, 52). The capacity of AI for the *de novo* design of biologically active molecules has the potential to enhance therapeutic efficacy (53). These technological advancements aim to refine MSC therapies by improving the identification of critical molecular components and drug targets, thus increasing the precision of drug development (54, 55). The collaboration of the pharmaceutical industry with AI firms to develop advanced platforms underscores the role of AI in simplifying the drug discovery process, necessitating rigorous clinical validation to ensure the efficacy and safety of these innovations (56).

#### 5.1.2 Leveraging AI for enhanced MSC therapy development

Integrating AI into MSC therapy research is a methodical strategy for exploring the detailed landscape of regenerative medicine. The ability of AI to analyze extensive data enhances the precision of identifying the molecular attributes of MSCs, which is fundamental in regenerative therapies aimed at repairing or regenerating tissues affected by conditions such as diabetes. The role of AI in automating the creation of therapeutic compounds represents a meaningful advancement toward improving regenerative treatment efficacy (57, 58). This synergy between AI and regenerative medicine research is poised to propel the development of new therapeutic strategies, potentially bringing significant benefits to patients with various chronic ailments.

### 5.2 ScRNA-seq in MSC therapies

The advent of scRNA-seq has enhanced the understanding and application of MSCs (59, 60). Despite the promising potential of MSCs in regenerative medicine and their success in preclinical models, clinical trials have often not met expectations, partly due to the heterogeneity of MSCs and inconsistent identification criteria. ScRNA-seq has bridged crucial gaps by enabling precise MSC characterization and biomarker identification and revealing gene expression heterogeneity within MSC subclusters (61). Such insights are invaluable for comprehending the functional diversity of MSCs and their roles in development, regeneration, and pathology. Furthermore, scRNA-seq helps to elucidate the dynamic transcriptional changes in MSCs during differentiation and the intricate signaling pathways regulating their key functions (20). This refined understanding, facilitated by evolving analytical methods and integration with histological research, promises more targeted MSC-based therapies, particularly in complex treatments such as islet transplantation for diabetes, contributing to personalized and effective interventions.

#### 5.2.1 Unveiling heterogeneity and potentials through ScRNA-seq

ScRNA-seq has improved MSC research, offering unprecedented insights into the isolation, identification, and classification of MSCs based on heterogeneity and subclusters (62). This technology allows for more precise characterization of MSCs, revealing the diversity of MSC subclusters and their specific molecular expression and functions (63). By revealing dynamic transcriptional changes and complex signaling pathways, scRNA-seq facilitates a deeper understanding of the roles of MSCs in development, regeneration, and pathology (64, 65). This in-depth knowledge is crucial for developing targeted MSC-based therapies, particularly for applications such as islet transplantation in diabetes treatment, by identifying MSC subpopulations with optimal therapeutic properties and guiding the refinement of clinical applications.

#### 5.2.2 Unveiling the biological function of MSCs through ScRNA-seq

The role of scRNA-seq extends beyond MSC differentiation to uncovering MSC-immune interactions. ScRNA-seq has illuminated the diverse ways in which MSCs modulate immune cells, from T cells to macrophages, through various cytokines and chemokines (66). This understanding is pivotal in diseases such as acute lung injury, where MSCs reduce proinflammatory immune cell infiltration and cytokine expression (67). Studies have also shown that MSCs influence macrophage behavior in lung fibrosis, indicating the potential of MSCs in immune modulation and tissue repair (68). This detailed understanding is crucial for the development of MSC-based treatments, providing fresh perspectives for personalized and effective diabetes care strategies.

## 6 Conclusion

This review underscores the significant impact of MSCs on islet transplantation for diabetes treatment. This finding highlights the role of MSCs in enhancing islet graft survival, modulating immune responses, and promoting angiogenesis and tissue repair, indicating their potential for use in diabetes management. Challenges such as MSC heterogeneity and the need for optimization in therapeutic applications are acknowledged, with advanced technologies such as AI and scRNA-seq offering promising solutions. The synergy between MSCs and islet transplantation is emphasized as a forward-looking approach to personalized, MSC-based interventions, setting a new direction in therapeutic strategies against diabetes.

## Author contributions

LM: Funding acquisition, Writing – original draft, Writing – review & editing. TW: Writing – review & editing. XW: Conceptualization, Funding acquisition, Writing – original draft, Writing – review & editing. ZP: Conceptualization, Writing – original draft, Writing – review & editing.
